# The full spectrum of clinical stages of psychosis at prison entry: prevalence and concurrent validity of symptom screening

**DOI:** 10.1007/s00127-024-02733-y

**Published:** 2024-07-30

**Authors:** Natalia Yee, Christie Browne, Prabin Chemjong, Daria Korobanova, Kimberlie Dean

**Affiliations:** 1 Justice Health and Forensic Mental Health Network, Roundhouse, Long Bay Complex, PO Box 150, Matraville, NSW 2036 Australia; 2 Discipline of Psychiatry and Mental Health, School of Clinical Medicine, University of New South Wales, Sydney, Australia; 3 Western Sydney Local Health District, Sydney, Australia

**Keywords:** Ultra high risk, First episode psychosis, Screening, At-risk mental state, Prisoners, Crime

## Abstract

**Purpose:**

Despite the high rates of psychotic disorders amongst people in prison, current prison mental health screening approaches have not included assessment of the full psychosis spectrum to capture those at-risk of an emerging psychosis as well as those with established illness nor assessed the concurrent validity of psychosis symptom screening.

**Methods:**

Using a clinical staging approach to establish the prevalence of Ultra High Risk (UHR), first episode of psychosis (FEP) and established psychosis (EP) groups, 291 adults entering custody in two prison reception centres in NSW completed a two-stage (screening and validation) interview process. The Comprehensive Assessment of At-Risk Mental States (CAARMS) was used to determine the clinical stages of psychosis and concurrent validity of symptom screening in identifying individuals on the psychosis spectrum was formally assessed.

**Results:**

Amongst men and women entering prison, almost one quarter (24.1%) met UHR criteria, 5.1% met the FEP threshold and 10.6% had an established psychosis. Those on the psychosis spectrum reported greater disadvantage across sociodemographic and justice factors. The presence of perceptual disturbance and paranoid beliefs emerged as the two best screening items for identifying those with an underlying psychosis spectrum illness.

**Conclusion:**

The prevalence of psychosis spectrum illness, including the UHR state, amongst those entering prison is high. Current prison mental health approaches should include screening for the presence of perceptual disturbances and paranoid beliefs to improve the detection of psychosis spectrum illness.

**Supplementary Information:**

The online version contains supplementary material available at 10.1007/s00127-024-02733-y.

## Introduction

The high rates of psychiatric morbidity among people in prisons compared to the general population is well-established. Approximately 4% of incarcerated adults worldwide have a schizophrenia-spectrum disorder, while 10% of men and 14% of women have depressive illnesses [1]. Rates of mental illness among people entering prison have been reported to be particularly elevated [2, 3].

Although the over-representation of psychotic illness and psychotic symptoms is well-recognised, few studies have considered psychosis beyond its unitary construct to include examination of the different clinical stages of the illness. The latter ranges from the at-risk stage where symptoms are incipient [also known as ‘Ultra-High Risk’ (UHR)], to the first episode of psychosis (FEP), and subsequently to the chronic and established stage of the illness. Identifying these different stages of illness is important given that they may be associated with different treatment needs [4–6]. The UHR stage is often thought to reflect a vulnerable mental state where symptoms are initial and mild, and such a stage *may* progress into a recognisable severe mental illness, such as psychotic or severe mood disorders [4]. Meanwhile, although the operational definition of FEP varies, it is most conventionally defined as the period between onset of frank psychotic symptoms and the start of antipsychotic treatment [7, 8].

Few previous studies of prison entrants have considered the early phases of the psychosis spectrum, with the first study undertaken in the United Kingdom by Jarrett et al. [9–11]. In their two-phase prison reception study, the authors used a modified version of the Prodromal Questionnaire-Brief Version (PQ-B) [12] and the Comprehensive Assessment of At-Risk Mental States (CAARMS) [13] to screen for UHR and psychotic symptoms in 891 adult men between the ages of 21 to 40 years who were entering custody. They found that 5% (*n* = 44) of prisoners screened met the UHR criteria on the CAARMS and 3% (*n* = 25) were in their FEP. The CAARMS was also used to establish UHR criteria in a sample of Irish young detainees (aged 16 to 20 years), with 23% of 171 newly committed young offenders found to be in the UHR phase [14]. In a recent Australian study examining the different clinical stages of psychosis in adult men referred to prison mental health services, the prevalence of UHR and FEP were found to be 6% in each of the two groups [15].

Prison entry presents an opportunity for early identification and intervention to address unmet mental health needs in a vulnerable and disadvantaged population. Despite the high rates of mental illness at prison entry, a significant proportion of mental illnesses, including psychotic disorders, remain undetected by existing prison screening processes [16, 17]. There is still no ‘gold standard’ for mental health screening in prison settings [18] and many international jurisdictions have had to rely on screening methods developed for the community instead or developed their own bespoke screening tools. Although some have shown promise, such as the Brief Jail Mental Health Screen (BJMHS) [19] and the Jail Screening Assessment Tool (JSAT) [20], none have included assessment of emerging psychosis or focused on establishing the concurrent validity of screening for psychosis specifically. Current approaches to prison mental health screening at reception may need to be modified to improve the detection of the psychosis spectrum among prisoners and to capture those at-risk or in the early stages of developing psychosis.

The present study aims to firstly establish the prevalence and characteristics (sociodemographic, clinical and criminal justice) of psychosis across the full spectrum of clinical stages (i.e., from UHR to FEP to Established Psychosis [EP]) in both adult men and women entering prison. The conventional definition of FEP, where symptoms meet illness threshold but remain untreated, is adopted for this purpose.The second aim of the study is to assess the concurrent validity of psychosis symptom screening in identifying the psychosis spectrum stages at prison entry.

## Methods

### Participants

The study sample comprised adult men and women who were recruited as part of the Prison Mental Health Screening (PMHS) study from the Metropolitan Reception and Remand Centre (MRRC) and Silverwater Women’s Correctional Centre (SWCC) between June 2016 and May 2018. These two maximum security facilities are the main reception centres for men and women entering custody in New South Wales (NSW). During the two-year recruitment period, a total of 7685 prisoners (6619 men and 1066 women) entered custody and 647 (377 men and 270 women) were randomly selected from the electronic register of prison entrants within 24 to 48 h of reception. Women were over-sampled to ensure a sufficient sample of women would be available for analyses stratified by sex. Of those randomly selected, 30 individuals were excluded for being too physically or mentally unwell to participate, having insufficient English language proficiency, lacking capacity to consent, or posing too great a risk to be safely interviewed. Of the remaining participants deemed eligible (*n* = 617), 60 (9.7%) declined to participate, 216 (35.0%) were unavailable for interview for various reasons (e.g., legal appointments, work, education or visit commitments, transferred to another prison or released) and 341 (55.3%) completed the baseline interview. A total of 291 individuals (205 men and 86 women) went on to complete both the baseline interview and subsequent validation interview and thus were included in the final analyses. Interviewed participants were reimbursed for the time contributed to completing the study interviews.

### Instruments and procedure

Each participant completed face-to-face interviews with a research officer (PC), a registered mental health nurse. The second validation interview was conducted within one week of the first interview. The health records (Justice Health and Forensic Mental Health Network [JH&FMHN] files) and criminal justice records (using the Corrective Services New South Wales [CSNSW] Offender Integrated Management System [OIMS]) for participants were also examined to obtain corroborative mental health and criminal justice information. A structured questionnaire was used in the baseline interview to obtain key sociodemographic information (e.g., age, birth country, marital status, legal status, employment status, First Nations background), clinical information (e.g., family history of mental illness, personality disorder diagnosis, history of head injury) and criminal justice information (e.g., offence type, victim type, age at first criminal justice system contact).

The Prison Mental Health Screening (PMHS) study (Dean, Browne, Korobanova, Chemjong, Yee & Spencer, submitted) tested the performance of a tool developed by research and clinical staff in response to the lack of a suitable existing tool. The PMHS tool (Supplementary file 1) was designed to identify those with potential mental health problems and/or self-harm risk requiring further assessment and management, and consisted of 12 main questions covering three key domains: a self-report psychiatric history questionnaire, a ‘key symptom’ questionnaire (incorporating selected questions from established screening tools) to detect both lifetime and current presence of key symptoms of major mental disorders, and a series of questions about past self-harm/suicidal behaviour and current cognitions. The tool itself took 20 to 30 min to administer. The psychosis spectrum items in the ‘key symptom’ domain were derived from Structured Clinical Interview for DSM for Axis 1 Disorders (SCID-1) [21] and Prodromal Questionnaire, 16-Item (PQ-16) [22] and were the focus of the concurrent validity component of the current study.

The PMHS was administered to participants during the baseline interview and, for the current study, questions from the psychiatric history section were used to aid determination of psychosis spectrum status. Specifically, participants were asked about their lifetime history of psychosis (i.e., *Prior to coming into prison have you ever had schizophrenia/bipolar disorder/drug-induced psychosis*?) and their lifetime history of antipsychotic treatment (i.e., *Ever treated for psychosis*?). A positive response to either question was assumed to indicate the presence of a lifetime history of psychosis, and the lifetime treatment question was used to distinguish between those who had untreated psychosis (i.e., FEP) and those with an established psychotic illness.

To screen for lifetime and current symptoms of psychosis, the PMHS tool ‘key symptom’ domain included six specific psychosis screening questions: a) *Have you ever heard things that other people couldn’t*,* such as noises*,* or the voices of people whispering or talking; OR had visions or saw things that other people couldn’t see?* (auditory and visual hallucinations); b) *Have you ever thought or felt that someone is going out of their way to give you a hard time*,* or trying to hurt you* (paranoia); c) *Have you ever felt that you were especially important in some way*,* or had special powers to do things that other could not do?* (grandiosity); d*) Have you ever felt as if your thoughts were being broadcast out loud so that other people could actually hear what you were thinking; or believed that someone could read your mind?* (thought broadcast); (e) *Have you ever felt that you’re not in control of your own ideas or thoughts or felt as though another person or force was interfering with your thoughts?* (thought interference); (f) *Have you ever seen special meanings in advertisements*,* shop windows*,* in the way things are arranged around you or received messages from the TV or the radio?* (ideas of reference). Those who endorsed any of these were also asked if the symptoms were current (i.e., present ‘in the last month’). Each of the six items were scored as present (rated ‘1’) or absent (rated ‘0’), enabling the creation of a total screening score out of 6. These screening questions were used in the current study to assess the extent to which such screening at prison entry can identify individuals within the psychosis spectrum.

To establish the clinical stages of psychosis, the CAARMS [13], a semi-structured interview schedule used to assess UHR (Ultra High Risk) status, was administered to all participants in the validation interview. To meet UHR criteria, only the Positive Symptoms subscale (encompassing Unusual Thought Content, Non-Bizarre Ideas, Perceptual Abnormalities, and Disorganised Speech) is needed. The instrument can also be used for identification of those meeting the threshold for a current psychotic episode, defined as the presence of frank psychotic symptoms lasting longer than one week. The CAARMS and its application within NSW prisons has been previously described in further detail [15].

The CAARMS, along with the psychiatric history questions included in the baseline interview, was used to group participants according to the following four clinical stages:

1. No Psychosis (CAARMS negative, no lifetime psychosis history).
2. Ultra High Risk or UHR (CAARMS subthreshold symptoms, no lifetime psychosis history).
3. First Episode Psychosis or FEP (CAARMS threshold for psychosis met, no past antipsychotic treatment).
4. Established Psychosis or EP (presence of lifetime psychosis history and treatment, CAARMS status further dividing this group into EP with current psychotic episode, EP with current residual symptoms, and EP in remission).

### Ethical review

The study was approved by the Justice Health and Forensic Mental Health Network Human Research and Ethics Committee (Ref: G185/14), the NSW Aboriginal Health and Medical Research Council Ethics Committee (Ref: 1137/15), and the Corrective Services New South Wales Ethics Committee (Ref: D16/139,081).

### Statistical analysis

Chi-square analyses were used to compare the four psychosis spectrum groups with regard to categorical dependent characteristic variables. ANOVA and Kruskal-Wallis tests were used for continuous dependent variables. Due to the oversampling of women, weighting was applied for all analyses – women were weighted to a factor of 0.33 and men had a weight factor of 1:00.

To obtain concurrent validity calculations, the UHR, FEP and EP groups were combined to form the ‘psychosis spectrum’ group (consisting of cases only) and the ‘no psychosis’ group formed the control sample. Separately, to determine whether the psychosis screening questions were better at identifying those above the diagnostic threshold, the FEP and EP groups were combined to form the ‘psychosis threshold’ group and the UHR and No Psychosis formed the ‘no psychosis threshold’ group. Concurrent validity psychosis screening items and a cumulative psychosis screening score were examined through calculation of sensitivity, specificity, positive predictive value (PPV) and negative predictive value (NPV) rates, as well as Receiver Operating Characteristic (ROC) and Area Under the Curve (AUC) values. All analyses were performed using the IBM SPSS Statistics for Windows, version 27.0 [23]. Any subgroup findings with five or fewer individuals were not reported to avoid potential re-identification.

## Results

### Prevalence of the clinical stages of psychosis amongst prison entrants

**Fig. 1:**
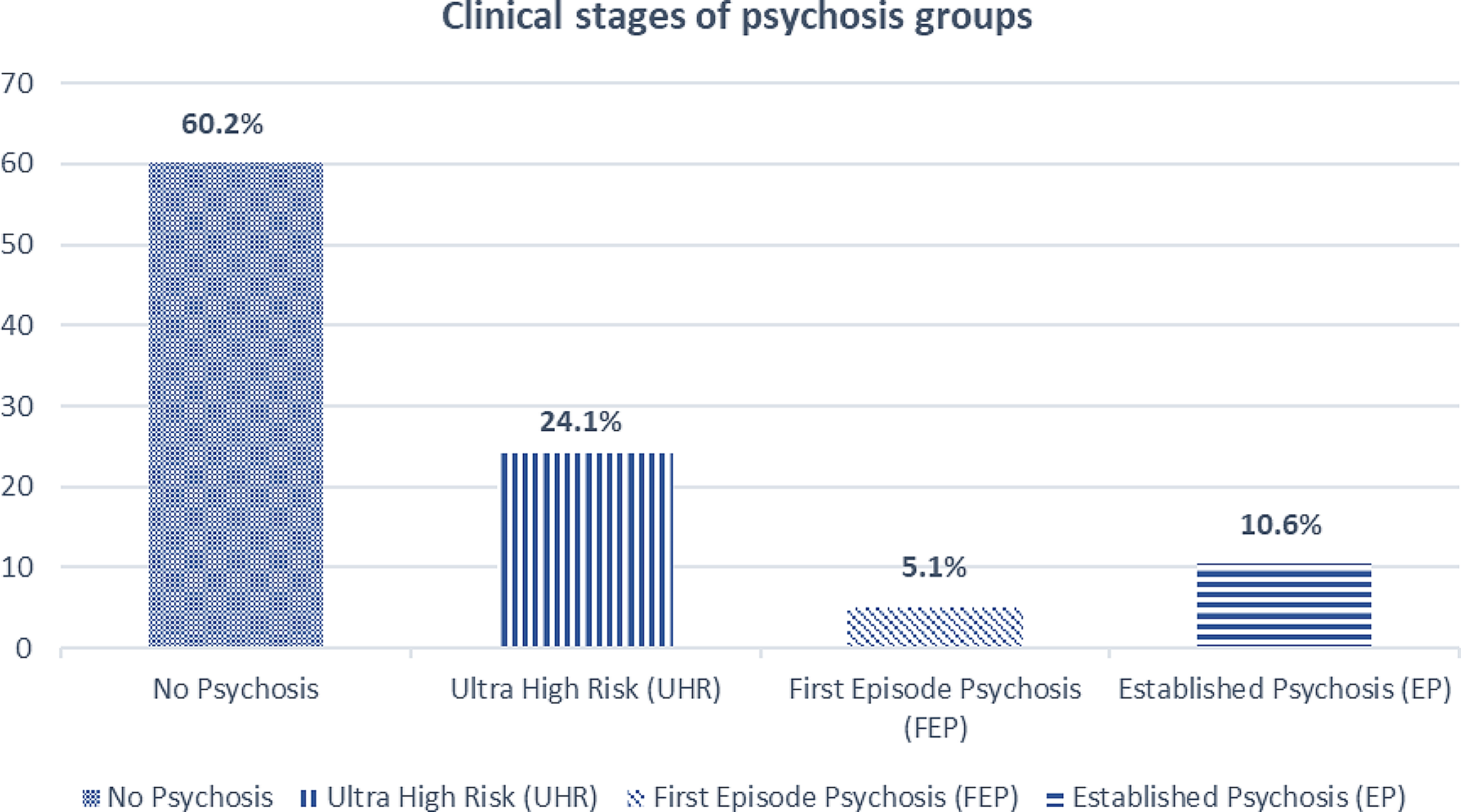
Prevalence of the clinical stages of psychosis at prison reception (*N*_weighted_= 233)

Four main groups were identified based on participants’ CAARMS profile and self-reported lifetime history of psychosis and antipsychotic treatment. As can be seen in Fig. 1, 60.2% of the weighted sample (*N* = 233) reported no previous history of psychosis and had no current psychotic symptoms. A further 15.7% of participants met the psychosis threshold (5.1% first-episode psychosis, FEP; 10.6% established psychosis) and almost a quarter (24.1%) of individuals reported subthreshold symptoms meeting UHR criteria. Depending on current symptomatology according to the CAARMS, those with an established psychosis (10.6%) could be further categorised as either being currently in remission (3.9% of total sample), having residual psychotic symptoms (3.3%) or suffering from a current psychotic (3.4%) phase of illness.

### Sociodemographic, clinical and criminal justice characteristics across the spectrum of clinical stages of psychosis

Participants were on average 34.7 years (*SD* = 11.0 years) at time of interview (Table 1) and the majority were male (87.6%), unemployed at the time of index offence (70.1%) and born in Australia (84.4%). Approximately one in four participants (23.8%) identified as having a First Nations background (i.e., Aboriginal and/or Torres Strait Islander Australians). More than half of the participants were on remand at time of interview (69.1%) for a non-violent index offence (59.1%) and the majority had been in custody before (72.5%). Just under half (45.5%) had a prior diagnosis of depression and 37.1% had prior anxiety diagnosis; a small proportion of participants (11.5%) had been diagnosed with a comorbid Axis II personality. Only 20.9% of participants in the current study were referred to the prison mental health service.

Chi-square analyses revealed that the relationship between stages of psychosis group and each of the following sociodemographic characteristics was statistically significant: First Nations status [*χ*^2^(3) = 7.80, *p* = .05], history of head injury [*χ*^2^(3) = 9.16, *p* < .05], unemployment at time of offence [*χ*^2^(3) = 15.31 *p* < .01] and history of childhood trauma [ *χ*^2^(3) = 27.11, *p* < .001]. Those in the FEP group were most likely to have a First Nations background (41.7%), a history of head injury (83.3%) and a history of childhood trauma (83.3%); meanwhile, those in the EP group were most likely to have been unemployed at time of offence (92.0%). The groups also differed regarding their incarceration history with those without psychosis being most likely to report being in custody for the first time (35.5%), *χ*^2^(3) = 12.84, *p* = .005. The EP group was most likely to be referred to prison mental health service (48.0%, *χ*^2^(3) = 13.61, *p* < .01).

**Table 1:** Weighted sociodemographic, clinical and forensic characteristics across the clinical stages of psychosis (*N*_weighted_ = 233)

	No Psychosis (*n* = 141)	UHR (*n* = 56)	FEP (*n* = 12)	Established Psychosis (*n* = 25)	Statistics
Age, *M (SD*)	35.62 (11.88)	32.25 (9.09)	32.78 (10.94)	36.16 (9.46)	*F* (3,229) = 1.526, *p* > .0*5*
Sex					
• Males • Females	126 (89.4%)	49 (87.5%)	9 (75.0%)	21 (84.0%)	*X*^2^ = 2.457 (3), *p* > .05
	15 (10.6%)	7 (12.5%)	3 (25.0%)	4 (16.0%)	
First Nations					
• Yes	25 (17.7%)	18 (31.6%)	5 (41.7%)	8 (32.0%)	*X*^2^ = 7.798 (3), *p* = .05
• No	116 (82.3%)	39 (68.4%)	7 (58.3%)	17 (68.0%)	
Education level < Year10					
• Yes	46 (32.6%)	26 (45.6%)	4 (33.3%)	13 (52.0%)	*X*^2^ = 5.328 (3), *p* > .05
• No	95 (67.4%)	31 (54.4%)	8 (66.7%)	12 (48.0%)	
Unemployed at I/O					
• Yes	86 (61.0%)	46 (82.1%)	9 (75.0%)	23 (92.0%)	*X*^2^ = 15.308 (3)**
• No	55 (39.0%)	10 (17.9%)	3 (25.0%)	2 (8.0%)	
Born overseas					
• Yes	25 (18.0%)	5 (8.9%)	1 (8.3%)	5 (20.8%)	Fisher’s exact = 3.331, *p* > .05
• No	114 (82.0%)	51 (91.1%)	11 (91.7%)	19 (79.2%)	
Childhood trauma					
• Yes	54 (38.6%)	41 (75.9%)	10 (83.3%)	13 (52.0%)	*X*^2^ = 27.111 (3)***
• No	86 (61.4%)	13 (24.1%)	2 (16.7%)	12 (48.0%)	
History of head injury					
• Yes	66 (47.1%)	32 (58.2%)	10 (83.3%)	17 (68.0%)	*X*^2^ = 9.162 (3)*
• No	74 (52.9%)	23 (41.8%)	2 (16.7%)	8 (32.0%)	
Legal status					
• Remand	102 (72.9%)	38 (67.9%)	6 (50.0%)	15 (60.0%)	*X*^2^ = 3.986 (3), *p* > .05
• Sentenced	38 (27.1%)	18 (32.1%)	6 (50.0%)	10 (40.0%)	
First custody					
• Yes	50 (35.5%)	12 (21.1%)	2 (16.7%)	1 (4.2%)	*X*^2^ = 12.842 (3)**
• No	91 (64.5%)	45 (78.9%)	10 (83.3%)	23 (95.8%)	
Index offence includes violence					
• Yes	56 (40.3%)	25 (45.5%)	5 (41.7%)	8 (33.3%)	*X*^2^ = 1.065 (3), *p* > .05
• No	83 (59.7%)	30 (54.5%)	7 (58.3%)	16 (66.7%)	
Referred to mental health					
• Yes	22 (15.6%)	12 (21.4%)	3 (25.0%)	12 (48.0%)	*X*^2^ = 13.611 (3)**
• No	119 (84.4%)	44 (78.6%)	9 (75.0%)	13 (52.0%)	
Lifetime depression					
• Yes	49 (34.8%)	34 (61.8%)	9 (75.0%)	14 (56.0%)	*X*^2^ = 17.798 (3)***
Lifetime anxiety					
• Yes	37 (26.4%)	31 (56.4%)	7 (58.3%)	11 (44.0%)	*X*^2^ = 18.413 (3)***
Comorbid Axis II personality					
• Yes	9 (7.2%)	9 (17.6%)	n/a	n/a	Fisher’s exact = 7.315*

* *p* < .05, ** *p* < .01, *** *p* < .001

Note sociodemographic, clinical and forensic variables (except for ‘index offence includes violence’) obtained from demographic questionnaire; cells with 5 or less count not reported

### Timing of criminal justice events across the spectrum of clinical stages of psychosis groups

**Fig. 2:**
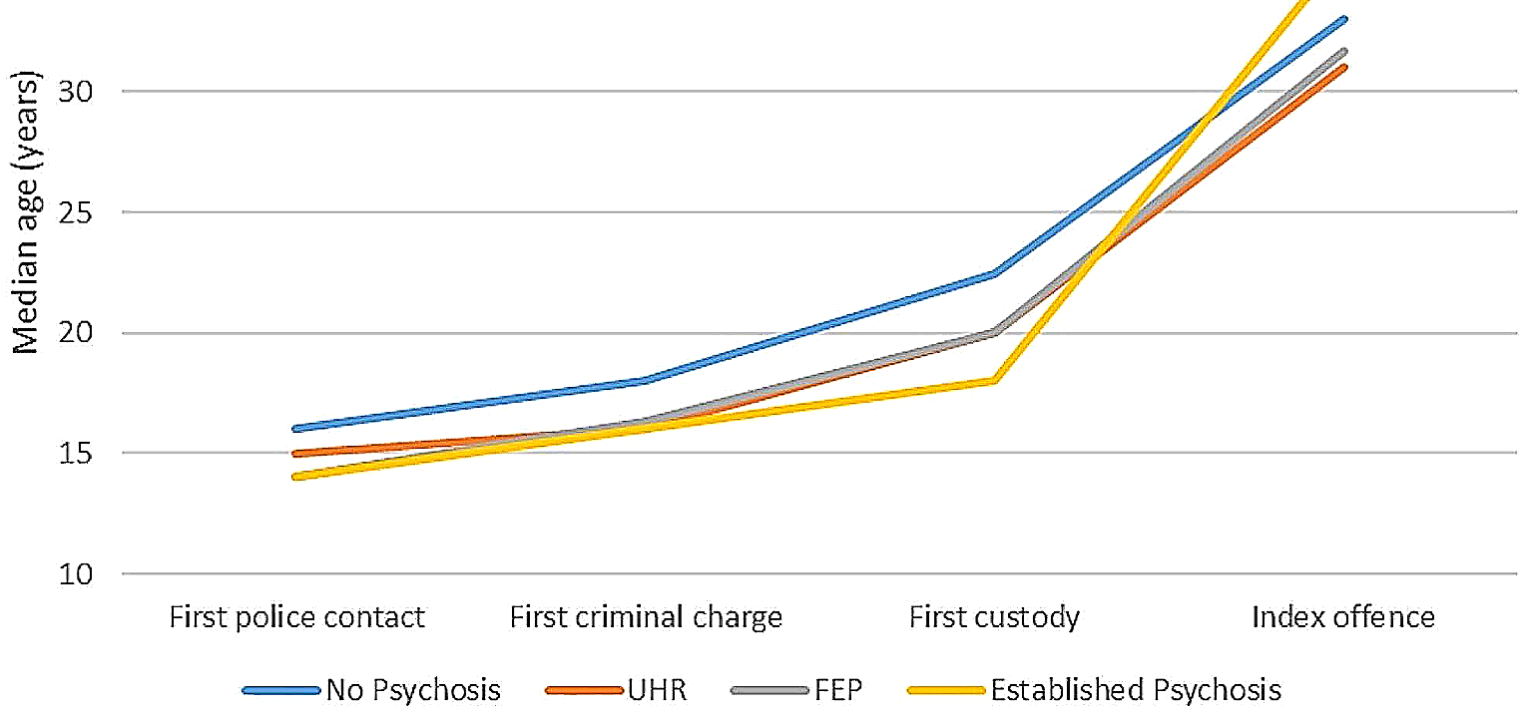
Median age for key offending events across the clinical stages of psychosis

Figure 2 displays the median ages associated with key criminal justice events for the four clinical stages of psychosis groups. Those without psychosis were older across the key criminal justice events, with the self-reported onset of criminal justice contact for this group occurring at median age of 16 years of age (*IQR* = 14–20), followed by first criminal charge two years later (*mdn* = 18.00, *IQR* = 15–23), first custody (*mdn* = 22.30, *IQR* = 18–32) and then the index offence (*mdn* = 33, *IQR* = 26–42). Meanwhile, the age of onset of first criminal justice contact appeared to coincide between the ages of 14 to 15 years for those in the psychosis spectrum groups (UHR, FEP and EP groups), with individuals across these groups experiencing their first criminal charge at the median age of 16 years old and first custody between the ages of 18 to 19 years. A Kruskal-Wallis H test revealed significant age differences between the clinical stages groups for age at first police contact [*H*(3) = 9.403, *p* < .05], age at first criminal charge [*H*(3) = 11.54, *p* < .01] and age at first custody [*H*(3) = 12.47, *p <* .01] but the groups did not differ in terms of their age at the time of offence leading to the current incarceration episode.

### Concurrent validity of psychosis screening at prison entry

For the assessment of concurrent validity in relation to distinguishing between those on the psychosis spectrum (including those meeting UHR criteria) from those not on the psychosis spectrum (Table 2), the best performing individual baseline symptom screening item was the presence of perceptual disturbance, with moderate sensitivity (60.9%), high specificity (89.9%) and an AUC of 0.76 (95% CI = 0.68–0.83). The presence of paranoid thoughts was the next best individual predictive item with a sensitivity of 78.5% and specificity of 69.3% (AUC = 0.74, 95% CI = 0.67–0.81).

Across the individual screening items, specificity tended to be higher than sensitivity, with the two most specific items being those measuring thought interference (‘*have you ever felt that you’re not in control of your own ideas of thoughts or felt as though another person or force was interfering with your thoughts?*’; 99.3% specificity) and grandiosity (‘*have you ever felt that you were especially important in some way*,* or had special powers to do things that others could not do?*’; 98.6% specificity). When the psychosis screening score was examined, endorsing any of the six screening items yielded a high sensitivity of 98.2% but low specificity of 29.1%. The best concurrent validity performance overall for the total screening score was a psychosis screening score of 2 or more (AUC of 0.86; 95% CI = 0.78–0.93), with a sensitivity of 71.0% and a high specificity of 92.9%.

**Table 2:** Concurrent validity for identifying psychosis spectrum status through screening at prison entry

	Endorsed item and screened positive, *n* (%)	Sens. (%)	Spec. (%)	PPV (%)	NPV (%)	AUC[95% CI]
*Psychosis screening questions*						
Q1: Have you ever heard things that other people couldn’t, such as noises, or the voices of people whispering or talking; OR had visions or saw things that other people couldn’t see?	56 (60.9)	60.9	89.9	80.0	77.6	0.76 [0.68–0.83]
Q2: Have you ever thought or felt that someone is going out of their way to give you a hard time, or trying to hurt you?	73 (78.5)	78.5	69.3	62.9	82.9	0.74 [0.67–0.81]
Q3: Have you ever felt that you were especially important in some way, or had special powers to do things that other could not do?	21 (22.6)	22.6	98.6	91.3	65.7	0.60 [0.52–0.69]
Q4: Have you ever felt as if your thoughts were being broadcast out loud so that other people could actually hear what you were thinking; or believed that someone could read your mind?	25(26.9)	26.9	97.9	89.3	67.0	0.62 [0.54–0.70]
Q5: Have you ever felt that you’re not in control of your own ideas or thoughts or felt as though another person or force was interfering with your thoughts?	33(35.5)	35.5	99.3	97.1	70.0	0.67 [0.59–0.75]
Q6: Have you ever seen special meanings in advertisements, shop windows, in the way things are arranged around you or received messages from the TV or the radio?	34 (37.0)	37.0	96.4	87.2	69.9	0.66 [0.57–0.74]
*Screening scores (sum of all six screening questions)*						
0	8(8.6)	8.6	36.9	8.2	38.0	0.21 [0.15–0.28]
≥ 1	85 (91.4)	91.4	63.1	62.0	91.8	0.77 [0.71–0.84]
≥ 2	66 (71.0)	71.0	92.9	86.8	82.8	0.86 [0.78–0.93]
≥ 3	43 (46.2)	46.2	97.1	91.5	73.1	0.73 [0.63–0.83]
≥ 4	27 (29.0	29.0	98.6	93.1	67.6	0.65 [0.55–0.75]
≥ 5	18 (19.4)	19.4	100.0	100.0	65.1	0.62 [0.52–0.72]
6	4 (4.3)	4.3	100.0	100.0	61.3	0.52 [0.42–0.62]

When those with above threshold psychotic illness were separated from those without threshold-level illness, sensitivity results increased across all the psychosis screening items (Q1 to Q6), as outlined in Table 3 below. A history of perceptual disturbance was again the best predictor screening item with an AUC of 0.84 (95% CI = 0.76–0.92) and high sensitivity (86.5%) and specificity (80%). The next best predictive item was the presence of paranoid thoughts with an AUC of 0.70 (95% CI = 0.61–0.80) and a high sensitivity of 83.8% but only a specificity of 56.3%. A psychosis screening score of 2 or more items again yielded the best concurrent validity performance, AUC = 0.87 (95% CI = 0.80–0.95), with a high sensitivity of 89.2% and moderate specificity of 78.2%. Overall, the predictive performance of the screening items and score appeared to be slightly, although not consistently, better for identifying above illness threshold psychosis than the wider spectrum of psychosis. However, on ‘post-hoc’ analysis, there was no significant advantage for concurrent validity for the first two psychosis screening questions considering positive responses to ‘either’ or ‘both’. This was true for predicting both the psychosis spectrum and psychosis threshold categories.

**Table 3:** Concurrent validity for identifying psychosis threshold (FEP and EP stages) through screening at prison entry

	Endorsed item and screened positive, *n* (%)	Sens. (%)	Spec. (%)	PPV (%)	NPV (%)	AUC [95% CI]
*Psychosis screening questions*						
Q1: Have you ever heard things that other people couldn’t, such as noises, or the voices of people whispering or talking; OR had visions or saw things that other people couldn’t see?	32 (86.5)	86.5	80.0	45.1	96.9	0.84 [0.76–0.92]
Q2: Have you ever thought or felt that someone is going out of their way to give you a hard time, or trying to hurt you?	31 (83.8)	83.8	56.3	26.5	94.9	0.70 [0.61–0.80]
Q3: Have you ever felt that you were especially important in some way, or had special powers to do things that other could not do?	13 (36.1)	36.1	94.9	56.5	89.0	0.66 [0.54–0.78]
Q4: Have you ever felt as if your thoughts were being broadcast out loud so that other people could actually hear what you were thinking; or believed that someone could read your mind?	11(30.6)	30.6	91.8	40.7	87.8	0.59 [0.47–0.71]
Q5: Have you ever felt that you’re not in control of your own ideas or thoughts or felt as though another person or force was interfering with your thoughts?	16 (43.2)	43.2	90.4	45.7	89.4	0.65 [0.54–0.77]
Q6: Have you ever seen special meanings in advertisements, shop windows, in the way things are arranged around you or received messages from the TV or the radio?	18 (48.6)	48.6	88.8	45.0	90.2	0.66 [0.54–0.78]
*Screening scores (sum of all six screening questions)*						
0	1 (2.7)	2.7	51.3	1.0	73.7	0.24 [0.16–0.31]
≥ 1	36 (97.3)	97.3	48.7	26.3	99.0	0.73 [0.65–0.81]
≥ 2	33 (89.2)	89.2	78.2	43.4	97.5	0.87 [0.80–0.95]
≥ 3	22 (59.5)	59.5	87.3	46.8	92.0	0.73 [0.61–0.85]
≥ 4	15 (40.5)	40.5	92.9	51.7	89.2	0.65 [0.53–0.78]
≥ 5	11 (29.7)	29.7	96.4	61.1	87.9	0.63 [0.51–0.76]
6	4 (10.8)	10.8	100.0	100.0	85.6	0.54 [0.41–0.66]

## Discussion

A high prevalence of psychotic illness, right across the spectrum of clinical stages, was identified in this study of men and women entering prison, including high rates of at-risk mental states (UHR) and first episode psychosis (FEP). Those on the psychosis spectrum reported greater disadvantage across sociodemographic and justice factors, and the presence of perceptual disturbance and paranoid beliefs emerged as the two best individual symptom screening items for identifying an underlying psychosis spectrum illness. While a high rate of psychotic illness and psychotic symptoms has previously been reported in an Australian prison context, this is the first time that the full spectrum of clinical stages of psychosis has been examined among individuals entering custody.

### Main findings – psychosis spectrum prevalence and profiles

The over-representation of psychotic illnesses in prisons is well-established with approximately 4% of incarcerated adults worldwide having a schizophrenia-spectrum disorder [1] and around 1 in 10 people in prison in Australia and New Zealand having evidence of current psychosis (Yee et al., submitted). Very few studies, however, have examined psychosis right across the illness spectrum in a prison-entry sample. In the present study, just over 10% adults entering NSW prisons reported having an established psychotic illness. Around half of this group (i.e., 1 in 20 adults entering custody) were experiencing their first episode of psychosis and had never been treated. This rate is slightly higher than the 3% FEP prevalence reported in a UK sample [9–11]. The prevalence of UHR cases found in the present study (24.1%) was similar to an Irish study of young people in detention [14] but higher than the 5% rate reported by Jarrett et al. [9] in the adult UK sample. These differences may relate to methodological variation between the two studies, including in relation to sample selection and in the use of the CAARMS. The latter was only administered to those who screened positive on the Prodromal Questionnaire in Jarrett et al’s [9] study, whereas all participants in the current study completed the CAARMS in the validation interview.

Compared to prisoners without psychosis, those on the psychosis spectrum (UHR, FEP and EP) had greater evidence of disadvantage. They were more likely to have a history of head injury, to be unemployed at time of offence, to have a history of childhood trauma, to have past diagnosis of depressive and anxiety disorders and a history of prior incarceration. People entering prison with evidence of psychotic illness were more likely to come from a First Nations background, supporting the notion that more effective mental health diversion for First Nations people may reduce their relative incarceration rates which are known to be alarmingly elevated. Although First Nations people represent about 3.8% of the total Australian population [24], almost a quarter (24%) of our sample identified as Aboriginal and/or Torres Strait Islander, highlighting the ongoing over-representation of this group in custody.

The psychosis spectrum groups were also found to be younger at the time of key criminal justice events compared to those without psychosis. Those with psychosis were not more likely to have been charged for a violent offence however. These findings are largely consistent with our earlier study of individuals with psychosis referred to prison mental health services [15]. The overlap in timing of key health and justice events suggests that the mid-adolescent period may present a critical window of opportunity for early identification and intervention to prevent both onset of serious mental illness and criminal justice contact. The greater social disadvantage, earlier exposure to the criminal justice system and experience of repeated justice contact for those with psychosis spectrum illness may be both a cause and consequence of the development of a serious mental illness.

### Main findings – concurrent validity of psychosis screening at prison entry

Six individual symptom-related items were used in the current study to screen for psychosis. When concurrent validity of the items was assessed, specificity tended to be higher than sensitivity across all the individual items, suggesting that the screening questions were more useful for screening ‘out’ those without psychosis than for screening ‘in’ those with psychosis spectrum illness, including those who were at-risk for psychosis. However, sensitivity improved when screening was validated against meeting the psychosis threshold (compared to the broader definition which included the UHR cases), confirming the challenges associated with identifying the attenuated and undifferentiated symptoms characteristics of the UHR stage [25, 26].

The presence of perceptual disturbance was the best item in terms of predicting psychosis spectrum status (AUC = 0.76, 95% CI = 0.68–0.83) followed by the presence of paranoid beliefs (AUC = 0.74, 95% CI = 0.67–0.81). This pattern was again observed in relation to predicting only the presence of illness above the psychosis threshold (AUC for perceptual disturbance 0.84 and paranoid beliefs 0.70). Perceptual abnormalities are common psychotic phenomenon [27, 28] and simple auditory and visual hallucinations are the most common symptoms among at-risk individuals too [29]. When the psychosis symptom items were combined to form a score out of 6, achieving a score of 2 or more demonstrated excellent validity in predicting both the psychosis spectrum (AUC = 0.86) and the psychosis threshold (AUC = 0.87).

There is no gold standard for prison mental health screening [17, 18]. Few screening validity studies have considered psychosis [30] but none have thus far examined the broader psychosis spectrum or the at-risk stage specifically. Often, it can be challenging to find the optimal balance between sensitivity and specificity in the prison context where there is a need to ensure that individuals are not missed by services or subjected to unnecessary intervention. This applies to screening for the psychosis spectrum amongst those entering prison, noting that the illness can have an insidious onset which is difficult to identify [31] or alternatively, appear ‘attenuated’ in the chronic stages due to residual or remitting symptoms.

In the current study, screening for the presence of two key common symptoms of psychosis – the presence of hallucinations and paranoid beliefs - may be the best brief approach to identifying individuals with psychotic illness or psychosis risk and thus should be incorporated into prison mental health screening tools. Following this, an effective second-stage mental health triage process may be established to reduce the burden on stretched prison mental health services and ensure that those most in need are referred on for more detailed mental health assessment and intervention. This would be an example of utilising the Screening, Triage, Assessment Intervention and Re-integration (STAIR) model, which has shown promise as a prison mental health screening approach. In this model, all people entering custody are screened but identification of ‘caseness’ occurs at the triage stage by trained mental health staff before onward referral of positive cases for specialised mental health intervention and service planning [32, 33]. In the current study, fewer than one-third of reception prisoners with psychosis spectrum symptoms were identified and referred to specialist prison mental health services using the standard screening approach, suggesting many people with psychosis would have missed out on optimal care and treatment.

### Strengths and limitations

Past research focusing on psychosis in prison has typically ignored the full spectrum of clinical stages of illness and identified prisoners with either current or lifetime established psychosis [1, 3, 34]. The main strength of the present study is that it is the first to examine all the different clinical stages of psychosis among adults entering custody. Additionally, the study included both men and women, whereas existing similar studies typically only include men/boys [9–11, 14, 15].

One of the main limitations of the present study was the small sample size, limiting some subgroup analyses. Although there was good response rate from those approached for participation, many potential participants were excluded due to their unavailability at time of interview (e.g., legal appointments, visits, transferred). Consequently, the prevalence rates for psychosis might have been underestimated in the present study. Additionally, the sample size of women was insufficient, despite their oversampling, to examine the key study aims stratified by sex.

The lack of past substance use information is another study limitation although the high prevalence of comorbid substance use disorders among prisoners is well-established [35]. On a broader level, the challenges associated with the UHR concept and its detection [25, 26] and the varying definitions of the FEP highlight the need for clinicians and services to consider how best to optimise early identification and early intervention of psychosis, including for First Nations people. Also, given that fewer than 30% of UHR cases transition to psychosis within 3 years [31, 36] and most studies on UHR and early psychosis focus on adolescents and young adults, the current study sample, with an older age range, is likely to include a significant proportion of UHR individuals who will not go on to develop a psychotic illness. Conversely, there may be a higher conversion rate among vulnerable and disadvantaged populations, such as prison populations, than is reported in community samples. Furthermore, the early stages of psychosis may be more prevalent amongst people coming into contact with the justice system prior to the age of 18 years and thus our study of adult prisoners was limited by not including this group. Future studies might undertake a similar approach with a younger cohort.

## Conclusion

The present study is the first to examine the full psychosis spectrum including the UHR, FEP, and established psychosis stages among adult men and women entering an Australian prison. High rates of psychosis across the clinical stages of illness were found and the promising concurrent validity findings for brief symptom screening of the presence of perceptual disturbances and paranoid beliefs at reception suggest that these items might usefully identify those who would benefit from more comprehensive mental health assessment. Our findings underscore the importance of adequate mental health resourcing in prisons to meet the treatment needs of those with serious mental illness, but also highlight the need to invest in early intervention programs in the community for ultra-high risk individuals, as well as to expand programs that divert eligible individuals with psychosis into mental health treatment as an alternative to custody. Future research might also focus on psychosis amongst First Nations people in prison and the relevant timing of illness onset, substance use onset, health service contact and justice system contact to better understand the relationship between severe mental illness and risk of criminal offending.

## Electronic supplementary material

Below is the link to the electronic supplementary material.

Supplementary Material 1

## Data Availability

No datasets were generated or analysed during the current study.
